# Understanding workplace violence against medical staff in China: a retrospective review of publicly available reports

**DOI:** 10.1186/s12913-023-09577-3

**Published:** 2023-06-20

**Authors:** Yumei He, Eleanor Holroyd, Jane Koziol-McLain

**Affiliations:** 1 Auckland University of Technology, Private Bag 92006, Auckland, 1142 New Zealand; 2 School of Clinical Sciences, Auckland University of Technology, Private Bag 92006, Auckland, 1142 New Zealand; 3 Centre for Interdisciplinary Trauma Research, Auckland University of Technology, Private Bag 92006, Auckland, 1142 New Zealand

**Keywords:** Workplace violence, Healthcare, China, Communication, Physician-patient relations, Trust, Patient experience, Management, Patient-centred care, Socio-ecological model

## Abstract

**Background:**

Workplace violence against medical staff in China is a widespread problem that has negative impacts on medical service delivery. The study aimed to contribute to the prevention of workplace violence against medical staff in China by identifying patterns of workplace violence, key risk factors, and the interplay of risk factors that result in workplace violence.

**Methods:**

Ninety-seven publicly reported Chinese healthcare violent incidents from late 2013 to 2017 were retrospectively collected from the internet and analysed using content analysis. A modified socio-ecological model guided analysis of the violent incidents focusing on risk.

**Results:**

Physical violence, yinao, or a combination of physical and verbal violence were the typical forms of violence reported. The findings identified risk at all levels. Individual level risk factors included service users’ unreasonable expectations, limited health literacy, mistrust towards medical staff, and inadequacy of medical staff’s communication during the medical encounter. Organisational level risk factors under the purview of hospital management included problems with job design and service provision system, inadequacies with environmental design, security measures, and violence response mechanisms within hospitals. Societal level risk factors included lack of established medical dispute-handling mechanisms, problems in legislation, lack of trust and basic health literacy among service users. Situational level risks were contingent on risk factors on the other levels: individual, organisational, and societal.

**Conclusions:**

Interventions at individual, situational, organisational, and societal levels are needed to systematically address workplace violence against medical staff in China. Specifically, improving health literacy can empower patients, increase trust in medical staff and lead to more positive user experiences. Organizational-level interventions include improving human resource management and service delivery systems, as well as providing training on de-escalation and violence response for medical staff. Addressing risks at the societal level through legislative changes and health reforms is also necessary to ensure medical staff safety and improve medical care in China.

**Supplementary Information:**

The online version contains supplementary material available at 10.1186/s12913-023-09577-3.

## Background

Widespread violence in the Chinese health sector has created serious consequences. High rates of depressive symptoms among doctors were reported and violence from patients, their families, or other visitors has been explicitly identified as the major cause [1–4]. Doctors in China, whether they have experienced or witnessed workplace violence have subsequently self-reported low morale, low motivation, and anxiety [5, 6]. Furthermore, the impact of workplace violence is affecting the provision of healthcare, with a hospital survey reporting that more than 28% of medical doctors choose protective practice [6].

Workplace violence in the health sector has been a global concern for decades [7–9]. The World Health Organization (WHO) defines workplace violence as “the intentional use of power, threatened or actual, against another person or against a group, in work-related circumstances, that either results in or has a high degree of likelihood of resulting in injury, death, psychological harm, mal-development, or deprivation” [10]. Despite efforts to address the issue, workplace violence against medical staff is still a concern in China [11, 12].

Workplace violence in the Chinese health sector can involve considerable personal injuries. In most cases, physicians are consistently reported as the target of attacks [13, 14]. This differs from what is reported in the international literature, which places nurses most at risk [8, 15]. Findings from several studies have revealed the connection between the occurrence of workplace violence and organisational factors such as workload, job satisfaction of doctors, and doctor-patient communication during consultation [16, 17]. In addition, miscommunication and insufficient communication have been explicitly identified as major risk factors for violence against physicians [17, 18].

There is an international body of epidemiological studies on violence in the health sector which typically identify risk factors associated with perpetrators, staff victims, and workplaces [1, 7, 9, 19], the relationship between organisational factors and the occurrence of workplace violence have been established [15, 20, 21]. Specific organisational factors such as poor management structures, excessive workloads, unjustified delay, long queuing, unhealthy interpersonal relationships, and negative staff attitudes can induce aggressive workplace behaviours [15, 21]. Stress from workload and time pressures are perceived as factors that increase risk of violence in the workplace [22]. A cycle between stress and violence as noted in international literature [23] seems to be evident in the Chinese health sector, which adds to the already high levels of work-related stress and anxiety. This can lead to widespread depressive symptoms among staff [24], which then further increases the risk of violence [25]. Consistent with the international literature, stressful working conditions, the diminished general wellbeing of medical staff, along with low job satisfaction, may also result in poor staff attitudes, which can impact negatively upon patient-physician interaction and increase the risk of violence [13, 15].

While international studies may help understand the problem in their specific context, they offer limited insight into how to address the problem in the Chinese context as workplace violence against medical staff in the Chinese context demonstrated some unique features. For example, a general climate of mistrust between patients and physicians in the Chinese context has been noted by researchers and reporters [26, 27]. Low government financial input in the public health system [28, 29], the unreasonable pricing of medical services and misplaced incentives have been identified as contributing to a climate of mistrust [26]. Problems with the patient complaint systems are seen to undermine patients’ trust in the medical institutions and the medical profession [26, 30].

A review of literature from China hypothesised that system level issues in the wider community are important factors in the causal chain for violence in the health sector [31–34]. Other factors previously identified as possible causes of health workplace violence include healthcare system characteristics, legislation, media coverage, law enforcement, and a general climate of mistrust between patients and physicians [26, 28, 29]. However, it was largely unknown what specific concerns are embedded within these factors, and what other unnamed factors may exist. Further, it was not known how external contextual factors impact upon the risk factors identified at the individual and situational levels.

Despite all that is known, there has not been study to systematically investigate the workplace violence against medical staff in the Chinese healthcare sector and how intervention should be developed systematically to address risk factors at different levels. Therefore, this study aimed to contribute to systematic violence prevention intervention in the Chinese health sector. Specifically, the research aimed to answer the following research questions: (1) What are the different patterns and risk factors of violent incidents against medical staff reported in the Chinese healthcare sector and (2) What are the key risk factors and interplay that contribute to violence against medical staff in the Chinese healthcare sector?

The study focused on yinao and physical violence against medical staff in China, in which mainly doctors (physicians), nurses and at times allied health professionals, were victims of violence. Yinao is a Chinese term referring to a unique form of violence in China, which features severe disruption of hospital operations in combination with both physical and verbal abuse that aims to gain substantial financial compensation from the hospital.

## Methods

Content analysis [35] of violent incident reports were used to understand the different patterns and risk factors of workplace violence against medical staff in China.

### Data source and collection

There is no readily available official data of violence in the health sector in China. Therefore, violent incidents used for analysis in the current study were drawn from internet search of mainstream and social media, including Wechat and Weibo. Collecting data from the internet has been recognised as a feasible data collection method and widely used in research [36, 37].

Publicly available reports of violent incidents during a five-year period from 2013 to 2017 against medical staff in China were collected, which predominantly covered yinao and severe physical violent incidents that caused injuries or at times medical staff’s deaths. Violent incidents from 2013 to 2016 were gathered using both Baidu and Google search engines with the following search terms in Chinese: 伤医事件 (violent incidents against medical staff) and 医闹 (yinao). In 2017, with Wechat emerging as a popular social media app, browser searching was supplemented with subscribing to major Wechat public accounts that kept track of violence incidents that happened, including China Medical PhD Network (中国医学博士联络站), yihuxun医护讯, and yishang (医殇). These WeChat public accounts provided a platform with which individual social media users could upload and report violent incidents in time or share reports of violent incidents from the mainstream media on the internet. Some violent incident reports were only briefly available due to various filters applied by local governments. Some violent incidents were only reported by mainstream media after being exposed on social media. The search sometimes resulted in a summary of violent incidents over a certain period, which usually provided key information for a violent incident that could help track down the original reports and was prepared by concerned interest groups.

### Inclusion and exclusion criteria

Violent incidents were included if they met three criteria: (1) medical staff were among victims of violence; (2) the report met veracity criteria and (3) the report included sufficient details. In Chinese language, the generic term “shangyi shijian” (伤医事件) can refer to any violent incident against medical staff. The term “medical staff” is used as equivalent to the Chinese term yi (医), the short form of yihu renyuan (医护人员). For this study, medical staff included doctors (physicians), nurses and allied health professionals who met medical service users on a professional basis or at a healthcare providing setting. The inclusion criteria did not specify any specific type of healthcare setting to be included or excluded.

Although the primary target population of the research was medical staff, data collection noted if others such as hospital management, security, or police, or other patients or visitors were documented as additional victims. The second criteria, veracity, meant that only violent incidents that had been reported as confirmed and verified by relevant authorities and institutions (e.g., local police, hospitals involved in the incident, or news reports) and gave specific source of information were included. The third criteria, sufficient details, meant that only cases that reported the following details regarding the event were included: location (both name and location of the hospital), time, perpetrator(s), victim(s), consequence, post-incident investigation and presumed antecedents or triggers of the violence.

Events were included in the database only once, though they may have been reported repeatedly over time and by a range of sources. When there were multiple versions of a violent incident, and reports from multiple sources, data were abstracted based on whether the report was from a reliable source and whether the report provided key verified details. In some cases, information was abstracted across multiple reports of the same incident to have as complete a picture as possible. For example, follow-up information was often provided in later reports of incidents.

**Fig. 1:**
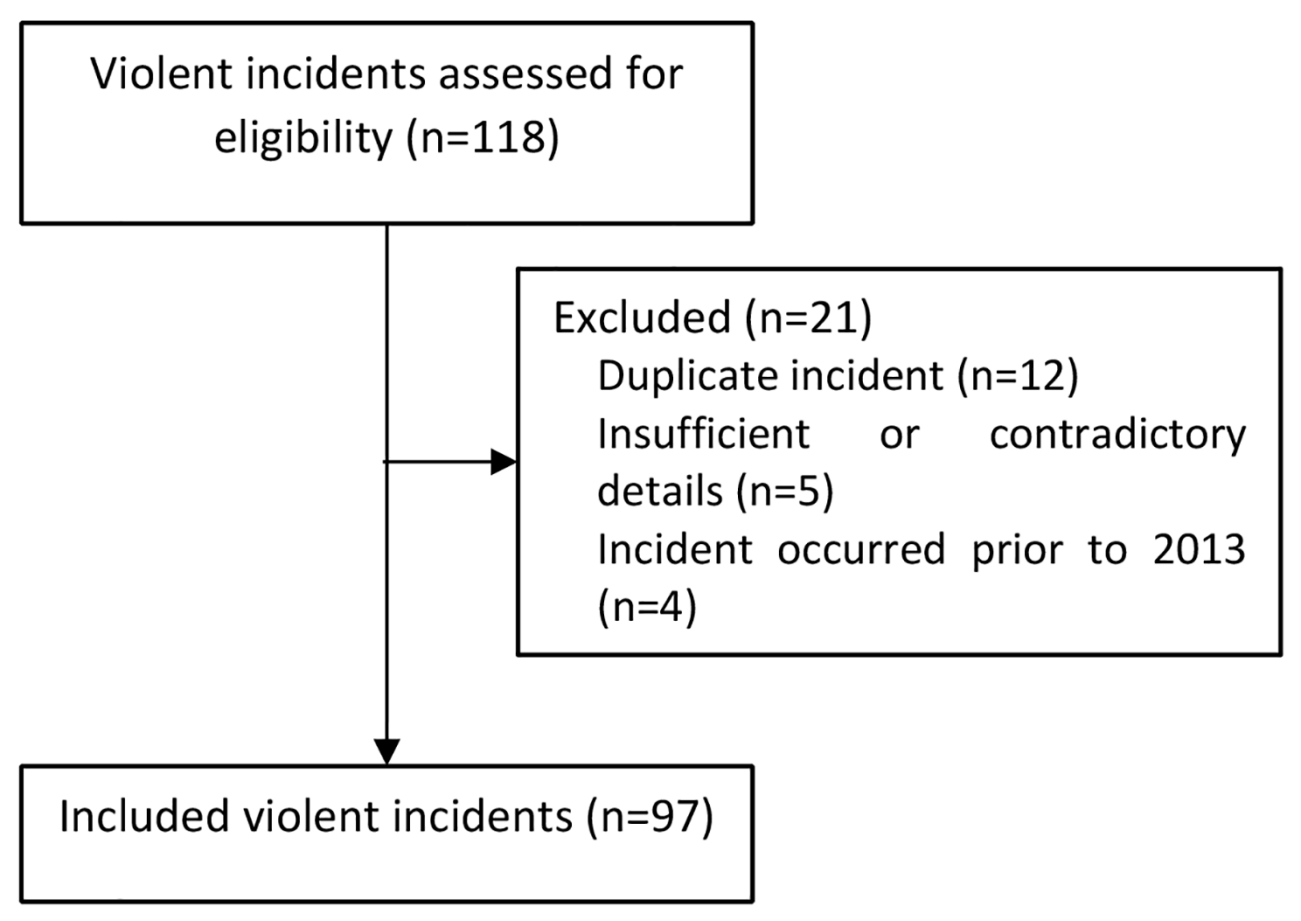
Selection of reported violent incidents

The search strategy resulted in 118 incidents to review. After applying the inclusion criteria, 97 unique violent incidents were identified (Fig. 1). Reporting of incidents in the public domain varied over time (from 9 in 2015 to 27 in 2017) due to changes in the use of social media, process of posting and deleting reports, and the Chinese context due to various filters applied at the local levels.

### Data management and analysis

Variables of interest were extracted from the selected reports for each violent incident using a standardised abstraction form (see Supplement file A). The variables of interest fell into several categories generally answering who, what, when, where, and why (see Supplement file B). The intent was to abstract data as close to what was reported as possible, to ‘let the data speak’. In some cases (particularly around the ‘why’), coding was based on referent context information. Specifying the variables and developing the code book (also referred to as a data dictionary) began with a draft variable list and coding instructions that were tested using a convenience sample of approximately one third of the violent incident reports. Any inconsistencies or ambiguity found in applying the code book resulted in the category being refined. After refinement, all collected violent incidents were coded by YH. The coding was done again weeks later to check for consistency and accuracy through intra-rater reliability, testing the reproducibility of coding [38]. The final coding resulted in consistent coding and all authors were satisfied with the code book.

Data (publicly available reports in Chinese language) were abstracted into a standardised form in English, coded and entered to SPSS software package (variables and code book were in English). In this process, translation was kept to a minimum. A small number of the data (n = 5) (Supplement file C) were managed with an extra step of translation and consultation: the original data in Chinese language were first abstracted into Chinese and then translated into English before analysis took place to enhance rigor.

Content analysis [35, 38] was employed with two levels of analysis. Firstly, a frequency query was run for all relevant variables to help identify possible dimensions or patterns of each variable. Importantly, the authors express caution in reporting frequencies as the sample of incidents was not considered representative of all violent incidents against medical staff in China, instead being limited to only those incidents that were reported (see limitation section). Secondly, factors associated with the violent incident reports were closely examined and interpreted to identify links between factors that fit at the different levels of the socio-ecological model. The focus of this second level of analysis was to interpret how these could contribute to the occurrence of violence towards medical staff both directly and indirectly.

### Framework of presenting risk factors

Data analysis and presenting of risk factors identified in the present study were guided with a modified socio-ecological model [39]. A system perspective is viewed as valuable in understanding workplace violence [22, 40] and in identifying health and safety risks [27]. The socio-ecological model [41–46] has been employed in studies on violence with variations of the model existing across different sectors [7, 10, 21, 47, 48].

## Results

Content analysis of the publicly available reports of 97 workplace violent incidents involving medical staff in China resulted in identifying key patterns. Reported violent incidents were typically physical violence, yinao or a combination of physical and verbal violence (Table 1). Interpreting the data resulted in identifying six key themes. Firstly, communication played a critical role in determining the outcome of the medical encounter (Table 2). In about 27% of the cases (n = 26), violence occurred while medical staff were communicating to ensure that service users complied with hospital rules and regulations. In another 14% of the cases (n = 14), violence occurred when the medical staff were carrying out communication over treatment plans or ward arrangements. In 6% of the cases (n = 6), the victim medical staff member was targeted while carrying out administrative tasks.

**Table 1:** Patterns of workplace violent incidents against medical staff in China reported publicly (n = 97)

Key variable (s)	Results
Primary violence type	Physical violence (n = 57, 58.8%), yinao (n = 18, 18.6%), combination of physical and verbal violence (n = 14, 14.4%) are the main forms of violence reported. Other (n = 8, 8.2%)
Incident location	Violence is a nationwide phenomenon, with distribution over different parts of the country, at different levels of the service delivery system and different types of hospitals, across private hospitals and public hospitals (97 incidents were reported at 26 different provinces, municipality, and special administration zone of the, ranging from 15 to 1 incident being reported (refer to Supplement file for details)
Main perpetrator	Patient family members account for a majority of perpetrators (n = 55, 56.7%). Patients of hospitals (current with registration or discharged) (n = 31, 32.0%). Both patients and visitors related to patients (n = 10, 10.3%).
Duration of incident occurrence	Most violent incidents lasted only briefly (n = 73, 75.3%). Yinao incidents aimed at substantial financial compensation from the victim hospital tended to last hours or days (n = 8, 8.2%).
Victim injury degree	Violence against medical staff in China can be very serious and can even cost the victim’s life (a total of 10 deaths in the sample from separate incidents)
Perpetrator gender	Males were reported to be the perpetrator in more cases than female: incidents involved male only (n = 57, 58.8%); incidents involved female only (n = 26, 26.8%); incidents with both perpetrators gender undocumented (n = 32, 32.9%)
Victim profession	Doctors were the predominant victims of the reported violence: doctors only being the victim (n = 58, 59.8%); nurses only being the victim (n = 26, 26.8%); both doctors and nurses being the victims (n = 6, 6.2%); others (n = 19, 19.8%)
Incident site	Doctor’s office or consultation room was the most vulnerable place where victims were attacked (n = 26, 26.8%)

**Table 2:** Interpreting situational risks involved in the medical encounter

Key Variable (s)	Results
What task may cause a risk What the violence was about: Resource shortage-related? Waiting-related? Trust-related?	Communication for compliance of rules and regulations (n = 26, 26.8%); Communication over treatment plans or ward arrangements which involved medical knowledge difficult for service users to understand (n = 14, 14.4%); Communication that should be more considerate for service users (n = 6, 6.2%); Not applicable (n = 51, 52.6%) Related to supply of facilities or resources (n = 9); Related to staff shortage (n = 9, 9.2%); Not related to resource supply or staff shortage (n = 78, 80.4%); unknown (n = 1, 1%) Related to having to wait briefly (n = 10, 10.3%); Related to having to wait due to technical or administrative reasons (n = 6, 6.2%); Related to having to wait to get access to resources (n = 2, 2%); Not related to waiting or not applicable (n = 79, 81.4%) Related to trust towards medical staff (n = 53, 54.6%); Not sure or hard to tell (n = 18, 18.6%); Not related to trust or not applicable (n = 21, 21.6%)
Presumption of perpetrator’s purpose	Violence was mainly spontaneous response to situation (n = 56, 57.7%), or for revenge or premediated to injure or harm (n = 19, 19.6%) or in some cases, to claim their justice or seek financial gain (n = 16, 16.5%)
Service users’ perspective reported explicitly & referent	Death of patient/unborn was hospital/medical staff’s fault (n = 24, 24.7%); Dissatisfaction over service delivery process, including medical staff’s communication or handling (n = 12, 12.4%); Dissatisfaction over treatment outcome, including no improvement or deterioration of medica condition (n = 9, 9.3%); Medical staff unable to give immediate attention or delay in providing treatment as requested (n = 9, 9.3%); Medical staff showing indifference/lack of understanding to patient’s suffering/situations, or respect to their feelings and background (n = 10, 10.4%)

Secondly, a low frustration tolerance and low trust towards medical staff were identified from the reported violent incidents (Table 2). It was found that communication situations that may appear to be neutral due to its administrative or informative content can trigger violence. This highlights service user lack of trust towards medical staff [42]. Over 54% of cases (n = 53) were found to be related to a lack of trust towards medical staff, and in another 5% of the cases (n = 5), violence was found to be related to a lack of trust towards both medical staff and medical dispute resolution authorities. The results suggest that trust is an important factor that impacts on the medical encounter and its outcomes.

Thirdly, in most reported cases, service users’ limited health literacy was noted as an issue (Tables 2 and 3). Limited health literacy of some service users prevented them from comprehending the unpredictable nature of their family member’s condition and necessary medical responses. It also led to unrealistic expectations that all illness can be cured, and death avoided through hospital treatment. Limited health literacy of service users can also be an important barrier for effective communication.

**Table 3:** Interpreting risks at organisational level

Key variables	Results and Learnings
Violence response system	Some mechanisms to respond to violence after harm has been caused or violence has been stopped (n = 42, 43.3%); No sign of established violence response mechanisms (n = 16, 16.5%); Some violence response mechanisms documented and helpful to minimise harm (n = 16, 16.5%); Some violence response mechanisms documented but not effective to be helpful in the case (n = 13, 13.40%); No applicable or unknown (n = 10, 10.3%)
Security measures evaluation: What went wrong in the reported case	Ineffective or slow security involvement without close security monitoring or inadequate security guards (n = 32, 33%); Undetected weapons or easy access to weapon, unmonitored visitor entry, visitors’ easy access within hospital, no panic button, and staff having no control over access to their working space (n = 29, 29.9%); Inadequate training and support provided to help staff to cope with violence effectively and minimise harm (n = 7, 7.2%); Little or no support provided to ensure staff’s safety during outbound assignment (n = 3, 3.1%); Not applicable or it goes beyond security measures /environment design of the hospital (n = 26, 26.8%)

A review of the reported incidents seemed to suggest that in the Chinese healthcare context service users tended to blame medical staff easily. Sometimes, even a change in a medical condition or no significant improvement of medical conditions could trigger anger and suspicion. For example, the perpetrator in one reported violent incident was angry because his child still had a fever after his first visit to hospital. Although the parents failed to follow the doctor’s instruction to get the child to take the medicine prescribed, the offender suspected the child’s lack of improvement was due to medical staff negligence. Some communication tasks while perceived as neutral due to their administrative or informational nature still carried the danger of being interpreted negatively by service users. Results of the analysis of variables in Table 2 revealed the importance of humanistic care during the medical encounters: concerns such as medical staff’s unpleasant words, attitudes, or lack of understanding during service delivery could trigger service users’ anger and result in violence. Table 2 summarised situational factors during the medical encounter that may triggered violence.

Fourthly, problems in service delivery management also created risks for violence against medical staff (Table 2). Analysis of violent incidents revealed risks manifested for individual medical staff and the situational context, impacted by hospital management. Specifically, problems with service provision processes, procedures, staffing, rules, and regulations led to service users to vent frustration and anger, which in turn created more friction and accordingly placed medical staff in more danger. Interestingly, the triggers were often “minute things” in the eyes of medical staff, which then escalated to anger and violence (Table 2).

Fifthly, safety measures such as monitoring visitor access within hospitals, visitors’ access to medical staff’s working and resting areas, and detecting weapons at hospital entry were found to be absent (Table 3). Importantly, training for medical staff on de-escalation and properly responding to violence and staying safe was found to be either absent or not effective. There was also no evidence of a standardised violence response system.

Sixthly, in the absence of established medical dispute resolution mechanisms, some service users resorted to violence (Table 4). Absence of credible channels for handling medical disputes interacted with the community lack of trust in medical staff and lack of health literacy, making communication with patients’ families extremely difficult (Tables 2 and 4). This was particularly evident in the violence incidents that followed the death of a patient.

**Table 4:** Interpreting risks at the societal level

Key variables	Results and Learnings
Service users’ efforts to resolve problems in non-violent ways before turning to violence	Service users directly resorted to violence after the death of the patient (n = 14, 14.4%); Service users have approached medical staff to communicate or seek shuofa (Chinese term for explanation and settlement) several times and not satisfied (n = 7, 7.2%); Violence occurred during communication with medical staff (n = 4, 4.1%); Not applicable (n = 61, 62.9%); Unknown (n = 4, 4.1%)
Was the alleged malpractice grounded? Attempt to try non-violent way to resolve problem	None of the alleged malpractice was supported by a medical assessment among the applicable cases (n = 35, 36.1%). Service users refused to have autopsy or medical assessment before resorted to violence (n = 12, 12.4%); Patient family refused to accept medical assessment results (n = 3, 3.1%); Patient family refused to accept the death of patient (n = 8, 8.2%) (Lack of established medical disputes handling mechanisms and lack of trust from medical service users towards medical dispute handling mechanism were apparent)
Local police involvement, local health authority involvement, local government involvement, external involvement	Inconsistencies or arguable external involvement from some key main stakeholders of government agencies in handling violent incidents observed (n = 22, 22.7%), which revealed problems in legislation and law enforcement: lack of clear legal references in handling medical disputes
Perpetrator consequence	Cost of violence for perpetrators too small to be adequate for both punishment and deterring violence against medical staff: no practical impact for perpetrator (n = 9, n = 9.3%), security or administrative arrest with or without fine (n = 37, 38.1%)

A summary of key risk factors identified from analysing the reported violent incidents is provided in Table 5. Risk factors were identified at different levels of the socio-ecological model: individual, situational, organisational, and societal levels. The situational level is not an independent level in the same sense as of individual, organisational and societal levels. The word “situation” as in situational level, situational factors can be interpreted both literally and as discussed by other researchers. Situational factors can include the interactions, interpretations during the medical encounter and the physical space involved that were relevant to the outcome of medical encounter [49]. Situational factors [50] or situational conditions [51] were contingent on risk factors at the other levels: individual, organisational, and societal.

**Table 5:** Summary of key risk factors identified from analysing reported violent incidents under the socio-ecological model

Individual level: Service user. Mistrust, limited health literacy, communication problems Medical staff. Compromised wellbeing; communication problems, including inadequate communication, lack of respect, inadequacy in communication skills
Situational level: Long waiting time, lack of trust between patient and physician, miscommunication/ insufficient communication
Organisational level: Showing characteristics of typical of high-risk working environment, including long waiting time, unpleasant physical environment, demanding job tasks due to job design and service provision management problems Inadequacy in hospital environment design and security measures taken, problems in handling patient discontent and complaints, absence of humanistic care
**Community and societal level**: Lack of trust between service users and medical staff, service users’ limited health literacy, absence of established credible medical dispute resolution mechanisms, problems with the external legal environment in handling medical disputes and handling violence towards medical staff

## Discussion

The findings gained in the current study revealed that the effective implementation of systematic violence incident reporting systems within hospitals would offer important evidence to inform prevention measures. The violent incident abstraction and variables used for data analysis of the present study may be helpful in designing a template for reporting future violent incidents. Systematic reporting can capture the situation of workplace violence against medical staff providing information to help understand antecedents of violence while offering useful insights for improving service delivery management, environment designs, and security measures for effective violence prevention.

Findings revealed a lack of consideration and respect shown towards service users as individuals and a failure to respect their emotional needs. This led to anger and resentment, and eventually violence towards medical staff. This is consistent with literature suggesting that addressing service users’ needs inclusive of the humanistic and technical aspects of care may be valuable in mitigating risk of violence [20, 31, 52–55]. This may require supporting medical staff to gain additional skills for effective communication [56–60]. Effective communication can facilitate humanistic care, contribute to quality care, and accordingly positive service user experience [61–65]. It would be difficult to expect a positive experience if medical staff only exhibited technical skills in the absence of effective communication during the medical encounter [66, 67]. Providing effective training to medical staff is therefore a necessary intervention strategy to address such a concern [68, 69]. Communication that conveys care, empathy, and responds appropriately to emotions can be learned and improved through effective training [70, 71]. Similarly, the environment can communicate care or indifference. In this study, service environments that were not user-friendly contributed to violence incidents. Literature suggests improving the service provision environment can improve patient satisfaction and outcomes [70], as well as medical staff job satisfaction [54, 72].

It is noted in most of the reported cases in which service users attributed the death of their loved one to the fault of medical staff, that trust from service users towards medical staff and medical service providing facilities was absent. The association between trust and violence in the Chinese context has been documented by Tucker et.al. [26]. Our findings support the intersection of mistrust, low frustration tolerance and low health literacy in contributing to violence against medical staff.

Service users’ inability to comprehend and accept death of patients and other adverse treatment outcomes was one of the main reasons for medical disputes and violence against medical staff in the Chinese context. Limited health literacy in this study is, therefore, defined as not knowing the limits of medical services and medical sciences, an inability in understanding the risk and uncertainty in medical services, an inability to understand the complexity of medical service, and a lack of acceptance of adverse treatment outcomes. Such a definition is not necessarily consistent with definitions in the literature [73]. However, it does capture the key reason identified behind service users’ misunderstandings, discontent, and even violence in the present study.

Consistent with such a definition, improving service users’ health literacy entails helping service users to comprehend the limitations of medical science, know the risks carried in medical services and exercise reasonability in accepting the adverse outcomes of the medical treatment received. Health literacy is viewed as an empowering mechanism [73, 74] that enables patients to play a more active role, take better control of, and manage their health. The enhanced sense of taking control can contribute to their trust in physicians [74, 75] and more positive experience [26, 76, 77]. Empirical evidence from the literature supports that improved health literacy can maximise communication and patient satisfaction when engaging with medical services [77, 78].

### Strengths and Limitations

The present study included only those violent incident reports that were publicly accessible on the internet. The number and types of violent incidents collected were likely subject to various filters, such as media preference and constraints of the socio-cultural context. The limited number of violent incidents collected made it impossible to further distinguish violent incidents based on its severity. In addition, the focus of the present study was on yinao and physical violence against medical staff in China.

A preliminary search identified a lack of reports of violent incidents resulting in emotional and psychological harm. Furthermore, psychological problems state and personal characteristics of individual involved are important factors contributing to violent incidents. The incident reports retrieved were not originally written to give a description of the psychological state or other details that enable analysis of psychological state. Such limitation inherited from a certain type of data suggest there is an important gap in our understanding of violence in the health workplace. Although the study identified pattens, key risk factors and possible interplays of risk factor, subject to limitations of data collection, there is not sufficient information to enable further exploration of interplay of risk factors. Future research would be valuable collecting data across multiple data sources.

Despite limitations, this study adds to the empirical knowledge of violence against medical staff in China, generating better understanding about patterns, key risk factors and their interplay. The findings revealed the pivotal role that hospital management can play in shaping service user experience and doctor-patient relationships. Specifically, many management aspects of hospitals, such as job designs, service provision procedures, environmental designs, security measures, and violence response mechanisms within hospitals were all found to have considerable room for improvement. The study also identified the need to integrate both service user experience and medical staff well-being into all aspects of hospital management.

## Conclusion

Focusing on a sample of reported violent incidents against medical staff from across China, at both private and public hospitals, we have gained an understanding of the pattern and key risk factors for the violence. We have highlighted the interplay between risk factors across the levels of the socio-ecological model. Understanding the interplay can guide prevention interventions.

Communication plays a critical role in determining the outcome of medical encounters. Limited health literacy, low frustration tolerance and low trust towards medical staff were also identified as risk factors on the individual level. Problems in service delivery management, absence of security facilities and measures, and lack of training for medical staff on properly responding to violence were found to be contributing factors on the organisational level. Additionally, the absence of established medical dispute resolution mechanisms and lack of trust in medical staff and limited health literacy among the population were identified as risk on the societal level. Overall, addressing these factors systematically is necessary to address situational risk factors, improve the quality of medical care and ensure the safety of medical staff. Specifically, societal, community and hospital management interventions are needed to create the environment to improve health literacy, foster trust and enhance communication to effectively address workplace violence against medical staff in China.

## Electronic supplementary material

Below is the link to the electronic supplementary material.

Supplementary Material 1

## Data Availability

The datasets used and analysed during the current study are available from the corresponding author on reasonable request.
