# Supplementary material for: Understanding workplace violence against medical staff in China: a retrospective review of publicly available reports
Source: BMC Health Serv Res. 2023 Jun 20;23:660. doi: 10.1186/s12913-023-09577-3 (PMC10283279; doi:10.1186/s12913-023-09577-3)
Supplement: Supplementary file 1 — Supplementary Material 1 [file 12913_2023_9577_MOESM1_ESM.docx]

Supplement file A: Violent Incident Abstraction Format

Record No.

Record ID:

File Name: VI

Original Link:
permanent link:

Source:

Date: (YY/MM/DD)

Time:

Duration of the incident:

Hospital Location:

Hospital Name:

Type of hospital: Comprehensive Western Medicine Hospital /Traditional Chinese Medicine Hospital/ Specialty Hospital

Level of hospital in Chinese:

Level of hospital: tertiary / secondary /community

Type of violence: Verbal violence (Y); Physical violence (Y); Yinao (N); Other

Perpetrator:

Number of perpetrator(s):

Perpetrator Gender:

Victim job title:

Victim gender:

Number of victim (s):

Victim’s specialty:

Victim professional title:

Incident Site:

Consequence (on victim and victim hospital)

Degree of Injury: Hospitalised: (Y/N); Rest/ Ward/ICU/ Death

Long term impact on victim:

Medical condition as reported:

Consequence (on perpetrator(s)’ side):

Mechanism of Injury (MOI):

Weapon used:

Trigger/antecedent as reported:

Reason given by perpetrator:

Detailed account of the incident:

Medical dispute involved? Yes/ No

Communication involved? Yes/No

Workplace environment:

Responses/helps available at site? Yes/No

Alarm/security camera in place? Yes/No

Source of help: self / Colleagues/ Security guards/passers-by

Follow-Up:

Police involvement: Yes/No

Local government involvement: Yes/No

Local authorities involvement: Yes/No

Notes:

**Supplement file B:**

**violent incident reports extraction categories**:

1) The type of incident (what)

2) Where the violent incident reported to occur

3) When the violent incident was reported to take place

4) Who were involved in the violent incidents

5) How the violent incident occurred

6) Antecedents of the violent incident; trigger and risk factors of violence during the medical encounter between medical staff and service users

7) Service users’ view the medical encounter and experience

8) External stakeholders involved in handling violent incidents

8) Security measures in place to ensure health and safety of medical staff at work; what was missing

9) Consequence and price of violence for victims based on follow-up reports of the incident

10) Consequence and price of violence for the perpetrator (s) based on follow-up report of the incident

**Supplement file C: Examples of Data Management Processed with Extra Translation and Consultation Procedure**

### Example 1

**Record No. VI1**

**Case ID:** VIYaan20170105

**File Name:** ABVIYaan20170105

**Link:** <http://finance.ifeng.com/a/20170105/15125370_0.shtml>
permanent link: <http://www.webcitation.org/6nNOKJ9Zz>
**Source:** [www.wccdaily.com.cn](http://www.wccdaily.com.cn) **(Huaxi Dushi Bao)**

**Date:** 2017/01/05 (YY/MM/DD)

**Time:** 1:28 am

**Duration of the incident:** No details available, being brief based on report

**Location:** Yaan, Sichuan Province

**Hospital:** Yaan People’s Hospital

**Type of hospital**: **Comprehensive Western Medicine Hospital** /Traditional Chinese Medicine Hospital / Specialty Hospital

**Level of hospital: tertiary** / secondary /community

**Type of violence:** Verbal violence (**Y**); **P**hysical violence (**Y**); Yinao (N); Other:

**Perpetrator:** Patient family

**Number of perpetrator**: 4

**Perpetrator Gender**: Male

**Victim:** Doctor

**Gender of victim:** Female

**Number of victim**: 1

**Victim’s specialty**: **Department of** Respiratory

**Accident Site:** Emergency Department

**Consequence (on victim and victim hospital):** victim was hospitalised for treatment with severe physical injury.

Degree of Injury: Hospitalised: (**Y**/N); Rest/ Ward/ICU/ Death

**Long term effect:** (disability/loss of workability/self-managing-ability)**:** NA

**Consequence (on perpetrator(s)’ side):** According to some informant, one perpetrator was under control of police and the other three were still at large. No comment from the police about that information.

**Trigger/antecedent as reported by victim or witness:** hospital unable to admit the patient as expected due to lack of bed

**Reason given by perpetrator:** The doctor used excuse to refuse request to provide service

**Mechanism of Injury** (MOI)**:** stabbing/beating/kicking/stepping

**Weapon used**: Legs

**Detailed account of the incident:** Doctor C was attending a consultation meeting at ED where the patient and family members requested hospitalization and getting treatment. Due to high demand of beds at Department of Respiratory, the doctor tried to explain to the perpetrators that there was no vacant bed at department of respiration but there would be a bed at the Emergency department to make it possible. The perpetrator interpreted the explanation as an excuse to refuse them service. The communication soon evolved into verbal conflicts and later ended up with four men chasing and beating the victim, including kicking, and stepping over the victim’s head. The victim was wounded all over her body and hospitalised. According to witness, the victim ended up with her finger tendon exposed and skin damaged.

**Medical dispute involves?** No

**Responses/helps at site:** Security guards

**Workplace environment:**

Alarm/security camera in place? Yes

**Self** / Colleagues/ Security guards/ Management

**Follow-up:**

**Police:** Police involved following a call and investigation followed.

**Local government:**

**Local authorities:**

### Example 2

**Record No. VI2**

**Record ID:** VIChangzhi20161123

**File Name: ABVI2**

**Link:** <http://cq.qq.com/a/20161123/019819.htm>
permanent link: <http://www.webcitation.org/6nNN39iUD>

**Source:** Beijing Youth Paper (www. ynet.com)

**Date:** 2016/11/22

**Time:** About 10am

**Duration of the incident:** Brief according to report

**Location:** Changzhi, Shanxi Province

**Hospital:** Heping Hospital Affiliated to Changzhi Medical Institute

**Type of hospital**: **Comprehensive Western Medicine Hospital** /Traditional Chinese Medicine Hospital / Specialty Hospital

**Level of hospital: tertiary** / secondary /community

**Type of violence: Verbal violence** (**Y**); **P**hysical violence (**Y**); Yinao (N); Other:

**Perpetrator:** Patient family

**Number of perpetrator**: 1

**Perpetrator Gender**: Male

**Victim:** Doctor

**Victim gender:** female

**Number of victim**: 1

**Victim’s specialty** **Department**: Infectious Diseases Department

**Accident Site:** Department of Geriatrics

**Consequence (on victim and victim hospital):** Victim was severely injured and was still in critical condition after emergency operation by the time of news report.

**Degree of Injury**: Hospitalised: (**Y**/N); Rest/ Ward/**ICU**/ Death

**Long term effect: (disability/loss of workability/self-managing-ability):** NA**,** victim’s life was still at risk at the time of report

**Consequence (on perpetrator(s)’ side):** the perpetrator turned himself in to the police

**Trigger/antecedent as reported:** The victim refused to give treatment in the way requested by the perpetrator, who had made the request based on information he obtained from internet.

**Reason given by perpetrator:** Same as above

**Mechanism of Injury** (MOI): **stabbing**/beating/kicking/stepping

**Weapon used**: Knife

**Number of attacks**: 9

**Detailed account of the incident:**

Doctor S from the Infectious Disease Department was stabbed nine times by a patient family with a knife, with one of the most serious stab piercing through her inferior vena cava and the right ventricular wall, which caused her to suffer from heart rapture, pericardial tamponade, and shock. After the emergency rescue operation, the victim was still in critical condition. The perpetrator’s 4-year-old daughter was an inpatient receiving treatment at the hospital for Hand-Foot and Mouth Disease (HFMD) and was getting better. The perpetrator searched online and obtained some information for HFMD and kept demanding doctor S to treat his daughter in the way described online, which was rejected by the doctor. Then the patient family then began to resist the doctor’s treatment plan after the refusal.

**Medical dispute involves?** No

**Communication involved? Yes**/No

**Workplace environment:**

**Responses/helps at site: No**

Alarm/security camera in place? **Yes**/No

**Self** / Colleagues/ Security guards/passersby

**Follow-up**: NA

### Example 3

**Record No. VI3**

**Record ID: ABVIGuangxi20150616**

**Original Link:** <http://news.163.com/15/0607/16/ARH64NM900014AEE.html>

Permanent link: [**http://www.webcitation.org/6mTLlQcx5**](http://www.webcitation.org/6mTLlQcx5)

**Source:** [www.thepaper.cn](http://www.thepaper.cn)

**Time:** 2015/06/16 (YY/MM/DD)

**Duration of the incident: NA,** brief

**Location: Nanning, Guangxi (Autonomy Region)**

**Hospital:** No. 1 Hospital Affiliated to Guangxi Medical University

**Level of hospital: tertiary**/secondary/community

**Type of hospital**: **Comprehensive Western Medicine Hospital** /Traditional Chinese Medicine Hospital / Specialty Hospital

**Type of violence:** verbal **/physical**/Yinao/ Other

**Perpetrator:** Former patient

**Perpetrator Gender**: Male

**Number of perpetrator**: 1

**Victim (s):** Doctor

**Victim Gender**: Male

**Number of Victim**: 1

**Victim Specialty**: Radiation Oncology department

**Incident Site**: Entry to elevator

**Consequence (on victim and victim hospital):** Injured with 30% to 35% of burns and ended up in ICU care. 72 hours after the rescue effort, the victim was still in a critical condition.

**Seriousness of Injury**: Hospitalised: (**Y**/N); Rest/ Ward/**ICU/** Death

**Long term effect:** (disability: loss of workability/loss of self-care-ability) possible

**Consequence (on perpetrator(s)’ side):** arrested following report

**Trigger/antecedent as reported:** dissatisfaction after treatment

**Reason given by perpetrator:**

**Mechanism of Injury** (MOI): stabbing/beating/kicking/stepping/**others: burning**

**Weapon used**: petrol and fire

**Trigger/antecedent as reported:** Dissatisfaction after the treatment

**Reason given by perpetrator:** Same

**Detailed account of the incident:** Doctor Q from Radiation Oncology department was poured petrol at the entry to the elevator and seriously burned by a former patient with nasopharyngeal carcinoma who was once treated by the doctor. The victim suffered from over 30% to 35% of third degree burns and ended up in ICU care. 72 hours after the rescue effort, the victim was still in a critical condition

**Medical dispute involved?** Yes/No/**Unknown**

**Legal procedure suggested by the hospital but rejected by patient/ family: NA**

**Workplace environment**

**Responses/helps available at site?** No, Victim was on his own.

Alarm/security camera in place? NA

**Follow-up:**

**Police:** Yes, after the violent incident for investigation.

### Example 4

**Record No: ABVI4**

**Record ID: ABVI4Yulin**

**Original Link:** <http://news.163.com/15/0607/16/ARH64NM900014AEE.html>
**Permanent link:** <http://www.webcitation.org/6nRzGLH76>

**Source:** [www.hsw.cn](http://www.hsw.cn) **(huashangwang)**

**Date:** 2015/06/05 (YY/MM/DD)

**Time: morning**

**Duration of the incident: Brief**

**Hospital Location:** Yulin City, Shanxi Province

**Hospital:** No.2 Hospital of Yulin City

**Type of hospital**: **Comprehensive Western Medicine Hospital** /Traditional Chinese Medicine Hospital/ Specialty Hospital

**Level of hospital: tertiary** / secondary /community

**Type of violence:** Verbal violence (**Y**); **Physical violence** (**Y**); Yinao (N); Other

**Perpetrator:** Patient and patient family

**Number of perpetrator**: 2

**Perpetrator Gender**: one female, one male

**Victim:** Doctor

**Victim gender:** Male

**Number of victim**: 1

**Victim’s specialty**: Department of Otorhinolaryngology

**Incident Site: consultation room**

**Consequence (on victim and victim hospital):** injured with eyeball cracked and risk of loss of vision permanently

**Degree of Injury**: Hospitalised: (**Y**/N); Rest/ **Ward**/ICU/ Death

**Long term impact on victim:** Possible loss of vision permanently and further treatment is needed

**Consequence (on perpetrator(s)’ side):** One perpetrator arrested for further investigation. No consequence for the other perpetrator, the mother who caused no injury.

**Mechanism of Injury** (MOI): stabbing/beating/kicking/stepping/**punching**

**Weapon(s) used**: medical history notebook, fist

**Trigger/antecedent as reported:** the patient’s request to jump the queue for treatment was rejected by the doctor (patient’s unreasonable request rejected by the doctor)

**Reason given by perpetrator:**

**Detailed account of the incident:** the patient together with his mother requested immediate consultation service from the doctor, L, claiming the patient was in a hurry to enter the national entrance exam, which was declined but the doctor. The patient’s mother got angry and threw the medical history notebook she was holding toward the doctor, which led to dispute between them. The patient, who was 16, punched the doctor with his fist. Before people could respond, the doctor’s left eye was injured.

**Medical dispute involved?** Yes/ **No/**unknown

**Legal procedure suggested by the hospital but rejected by patient/ family?** Yes/No/NA

**Communication involved? Yes**/No

**Unreasonable request being rejected**? **Yes**/No

**Motivation** of violence:

**Workplace environment:**

**Responses/helps available at site? Yes**/No

Alarm/security camera in place? **Yes**/No

**Self** / Colleagues/ **Security guards/bystanders**

**Follow-Up:**

**Police: Yes**/No

Police involved following a call and investigation followed.

**Local government: NA**

**Local authorities: NA**

### Example 5

**Record No. VI8**

**Record ID:** VI8Laigang

**File Name:** ABVI8

**Original Link:** <http://news.xinhuanet.com/legal/2016-10/05/c_1119664581.htm>
**Permanent link**: <http://www.webcitation.org/6oKjT6qde>

**Source:** [www.bjnews.com/cn](http://www.bjnews.com/cn) **(xinjingbao)**

**Date:** (YY/MM/DD) 2016-10-03

**Time:** morning, about 10 am

**Duration of the incident:** Brief

**Hospital Location:** Laifu, Shandong Province,

**Hospital:** Laigang Hospital

**Type of hospital**: Comprehensive Western Medicine Hospital

**Level of hospital: tertiary**

**Type of violence: Physical violence** (**Y**);

Perpetrator: Patient’s father, C

**Number of perpetrator**: 1

**Perpetrator Gender**: Male

**Victim:** Doctor Li

**Victim gender: M**ale

**Number of victim**: 1

**Victim’s specialty**: Department of Paediatrics

**Incident Site: doctor’s office**

**Consequence (on victim and victim hospital)**

**Degree of Injury**: **Death**

**Long term impact on victim:** NA

**Consequence (on perpetrator(s)’ side): arrest**

**Mechanism of Injury** (MOI): **chopping 15 times, 12 in head**

**Wounds: 15**

**Weapon used**: A chopping knife and a sharp knife

**Trigger/antecedent as reported: death of patient, who was new-born**

**Reason given by perpetrator: revenge**

**Detailed account of the incident:** The perpetrator’s new-born baby developed neonatal septicemia and pulmonitis, and died two days after birth due to rapid deterioration of her condition. Doctor Li who was the doctor on duty working at the ICU at Paediatrics Department communicated once with the patient family on the patient’s medical condition. After the death of his daughter, the perpetrator failed to reach a reconciliation agreement with the hospital, and he decided to take a revenge. On 3 October 2017, the perpetrator arrived with a backpack, carrying a chopping knife and a sharp knife. The victim Doctor L who had been working on his night shift was chopped 5 times by the perpetrator with the chopping knife in the doctor’s office. The victim died after rescue efforts in the afternoon.

**Medical dispute involved? Yes**/ No

**Communication involved? Yes**/No

**Workplace environment:**

**Responses/helps available at site?** Yes**/No**

Alarm/security camera in place? **Yes**/No

**Self** / Colleagues/ **Security guards/passers-by**

**Follow-Up:**

**Police: Yes/**No

Police involved following a call and investigation followed. The perpetrator was under arrest.

**Local government: NA**

**Local authorities: NA**

Guojia weijiwei, Ministry of Public Security sent officers to oversee the investigation of the case. The case was classified as a Serious Health professionals-related case (Zhongda Sheyi anjian).
